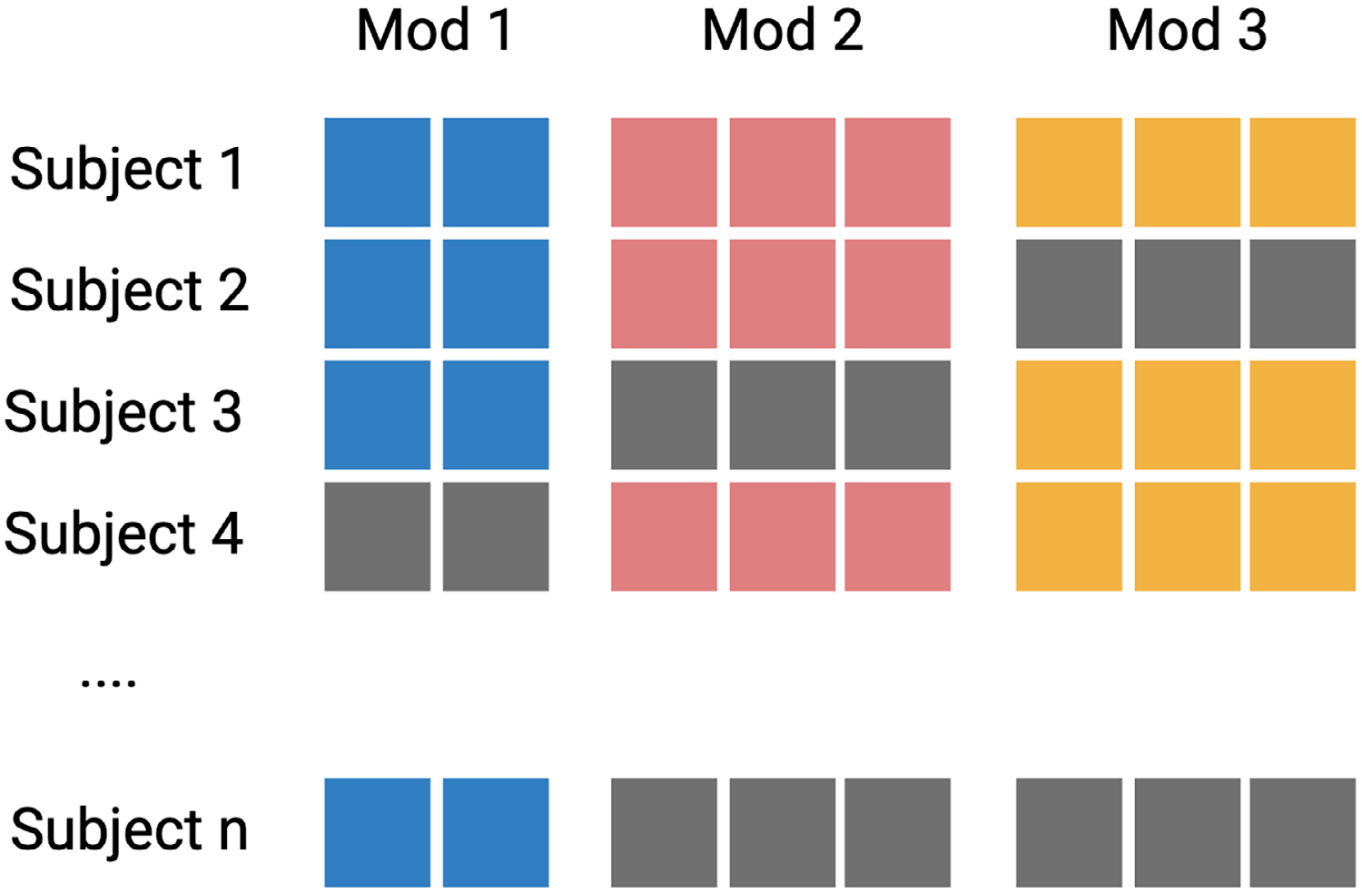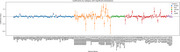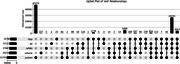# Double Machine Learning for Block‐Missing Data: Integrative Analysis of Plasma Biomarkers and Hippocampal Atrophy in Alzheimer's Disease Progression

**DOI:** 10.1002/alz70856_104028

**Published:** 2025-12-26

**Authors:** Yue Yang, Ting Li, Borui Tang, Tengfei Li, Hongtu Zhu

**Affiliations:** ^1^ University of North Carolina at Chapel Hill, Chapel Hill, NC, USA; ^2^ Shanghai University of Finance and Economics, Shanghai, Shanghai, China

## Abstract

**Background:**

Alzheimer's disease (AD), the predominant cause of dementia, is characterized by progressive cognitive deterioration, significantly impacting public health due to the absence of curative treatments. The insidious progression from asymptomatic stages to full‐blown dementia underscores the urgency of developing predictive tools for early detection. Recent advancements in proteomics and neuroimaging have identified potential biomarkers and structural brain changes associated with the disease, offering new pathways for understanding and potentially intervening in its progression.

**Method:**

We designed a novel double machine learning framework to de‐bias the analysis of complex block‐missing multi‐omics data. This approach enabled a comprehensive evaluation of the causal relationships between plasma biomarkers (GFAP, NEFL, GDF15, LTBP2) and hippocampal atrophy in the context of Alzheimer's disease progression. Our methodology combines rigorous statistical analysis with state‐of‐the‐art machine learning techniques to ensure robust and reproducible findings.

**Result:**

We identified multiple significant protein biomarkers and neuroimaging features associated with Alzheimer's disease (AD). The most prominent effects emerged in three protein biomarkers—GFAP, NEFL, and CST5—implicating astroglial activation, axonal injury, and inflammatory pathways. Functional connectivity disruptions (e.g., Net_Nodes_PC9, Net100_Pair21_38) underscored large‐scale network alterations, while additional proteins (BCAN, IGF2R, IGFBP3, FCRL5, PDGFC, ERP44, TNFRSF10A) indicated metabolic and immune‐related mechanisms. White matter integrity measures from diffusion MRI (RD_PTR_V3, FA_SFO_V5, AD_SCC_V2, FX.FA, FA_FX_V1, MD_SCC_V2) revealed microstructural damage in tracts tied to cognitive and memory functions. Lastly, hippocampal subfield changes (HippSubfieds_20, HippSubfieds_21, HippSubfieds_71) further highlighted the hippocampus's central role in AD pathology.

**Conclusion:**

By providing a comprehensive analysis of the key biomarkers and neuroanatomical changes, our study advances the understanding of Alzheimer's disease progression and opens avenues for the development of targeted treatments.